# Supplementary material for: Pathway metabolite ratios reveal distinctive glutamine metabolism in a subset of proliferating cells
Source: Mol Syst Biol. 2025 Jun 5;21(8):4. doi: 10.1038/s44320-025-00099-0 (PMC12322234; doi:10.1038/s44320-025-00099-0)
Supplement: Supplementary file 1 — Table EV1 [file 44320_2025_99_MOESM1_ESM.docx]

**Table EV1: Cell line details**

| **Cell line** | **Cancer type** | **Tissue type** | **Culture media** | **Overlap with DEMETER (Yes/No)** | **Overlap with Project Score**  **(Yes/No)** | **Overlap with Prism**  **(Yes/No)** | **Overlap with GDSC2**  **(Yes/No)** |
| --- | --- | --- | --- | --- | --- | --- | --- |
| MCF10A | Breast | normal | DMEM F12 + horse serum + EGF + hydrocortisone + insulin | N | N | N | N |
| MDA-MB-231 |  | tumour | DMEM HG | Y | Y | Y | Y |
| BT474 |  | tumour | RPMI | Y | N | Y | Y |
| MDA-MB-468 |  | tumour | RPMI | Y | Y | Y | N |
| MCF7 |  | tumour | DMEM HG | Y | Y | Y | Y |
| BT20 |  | tumour | RPMI | Y | N | N | Y |
| MDA-MB-134 |  | tumour | RPMI | N | N | N | Y |
| PNT1 | Prostate | normal | RPMI | N | N | N | N |
| PNT2 |  | normal | RPMI | N | N | N | N |
| C4-2B |  | tumour | RPMI | N | N | N | N |
| PC-3 |  | tumour | RPMI | Y | N | Y | Y |
| 22Rv1 |  | tumour | RPMI | Y | Y | Y | Y |
| LNCaP |  | tumour | RPMI | Y | Y | Y | Y |
| DU145 |  | tumour | RPMI | Y | Y | N | Y |
| CWR-AD1 |  | tumour | RPMI | N | N | N | N |
| CWR-D567 |  | tumour | RPMI + 10%CCS | N | N | N | N |
| VCAP |  | tumour | DMEM HG | Y | N | N | N |
| V16D |  | tumour | RPMI | N | N | N | N |
| MR49F |  | tumour | RPMI + 10$\mu$M enzalutamide | N | N | N | N |
| MR42D |  | tumour | RPMI + 10$\mu$M enzalutamide | N | N | N | N |
| AML12 | Liver | normal | DMEM/F12 | N | N | N | N |
| PH5CH8 |  | normal | DMEM HG | N | N | N | N |
| HEPG2 |  | tumour | DMEM HG | N | N | N | N |
| HUH7 |  | tumour | DMEM HG/1xGlut | N | N | Y | N |
| SKHEP1 |  | tumour | DMEM HG | Y | N | Y | Y |
| HEPA1-6 |  | tumour | DMEM HG | N | N | N | N |
| HUE-T | Endometrial | normal | Phenol-free DMEM/F12 | N | N | N | N |
| MAD11 |  | normal | DMEM HG | N | N | N | N |
| Ishikawa |  | tumour | DMEM HG | N | N | Y | Y |
| MFE296 |  | tumour | DMEM HG | N | N | Y | N |
| MFE319 |  | tumour | DMEM HG | N | N | Y | N |
| AN3CA |  | tumour | MEM | N | N | Y | N |
| RL952 |  | tumour | DMEM HG | Y | Y | N | N |
| KLE |  | tumour | DMEM/F12 | N | Y | N | Y |
| HEC1A |  | tumour | DMEM HG | Y | Y | Y | Y |
| U251 | Glioblastoma | tumour | DMEM HG | Y | Y | Y | Y |
| U87 |  | tumour | DMEM HG | Y | Y | N | Y |
| HPDE | Pancreatic | normal | Keratinocyte-SFM | N | N | N | N |
| PANC1 |  | tumour | DMEM HG | N | N | Y | N |
| MIAPACA-2 |  | tumour | DMEM HG | Y | Y | Y | Y |
| BXPC3 |  | tumour | RPMI + 2mM L- Glutamine | Y | Y | Y | Y |
| ASPC1 |  | tumour | RPMI + 2mM L- Glutamine | Y | Y | Y | Y |
| A2780 | Ovarian | tumour | RPMI | N | Y | Y | Y |
| OVCAR3 |  | tumour | RPMI | N | Y | Y | Y |
| SKOV3 |  | tumour | RPMI | Y | N | Y | Y |
| A375 | Melanoma | tumour | RPMI | Y | Y | Y | Y |
| HT144 |  | tumour | RPMI | Y | N | Y | Y |
| WM266.4 |  | tumour | RPMI | Y | N | Y | N |
| DO4-M1 |  | tumour | RPMI | N | N | N | N |
| A549 | Lung (NSC) | tumour | F12K | Y | Y | Y | Y |
| NCI-H226 |  | tumour | RPMI | N | Y | Y | Y |
| Detroit562 | Head and Neck | tumour | DMEM/F12 | N | Y | Y | Y |
| SCC4 |  | tumour | DMEM/F12 | Y | Y | N | Y |
| HT29 | Colorectal | tumour | DMEM HG | Y | Y | Y | Y |
| HCT116 |  | tumour | DMEM HG | Y | Y | Y | Y |
| SW-48 |  | tumour | DMEM HG | Y | Y | Y | Y |
| SW-1417 |  | tumour | DMEM HG | Y | N | N | Y |
