# Supplementary material for: Pathway metabolite ratios reveal distinctive glutamine metabolism in a subset of proliferating cells
Source: Mol Syst Biol. 2025 Jun 5;21(8):4. doi: 10.1038/s44320-025-00099-0 (PMC12322234; doi:10.1038/s44320-025-00099-0)
Supplement: Supplementary file 2 — Table EV2 [file 44320_2025_99_MOESM2_ESM.docx]

Table EV2: Overlapping cell lines and cluster membership identified using pathway-ratio analyses.

| **Cluster #** | **Ours** |  | **Shorthouse** | **Cluster #** |
| --- | --- | --- | --- | --- |
| **1** | MCF7 |  | MCF7 | **4** |
| **3** | **NICH226** |  | **NICH226** | **4** |
| **3** | **A549** |  | **A549** | **4** |
| **3** | DU145 |  | MIAPACA2 | **4** |
| **3** | BT20 |  | BXPC3 | **1** |
| **3** | **OVCAR3** |  | **OVCAR3** | **1** |
| **3** | **PC3** |  | **PC3** | **1** |
| **4** | MDAMB468 |  | MDAMB468 | **2** |
| **2** | MIAPACA2 |  | BT20 | **2** |
| **2** | HCT116 |  | HCT116 | **5** |
| **2** | **HT29** |  | **HT29** | **3** |
| **2** | **MDAMB231** |  | **MDAMB231** | **3** |
| **5** | **ASPC1** |  | **ASPC1** | **3** |
| **5** | **PANC1** |  | **PANC1** | **3** |
| **5** | BXPC3 |  | DU145 | **3** |

Bold cells indicate pairs of cell lines clustered together in both ours and Shorthouse *et al.* (2022). Rows are ordered to align common cell lines between the two datasets without splitting members of a cluster.
