# Supplementary material for: Pathway metabolite ratios reveal distinctive glutamine metabolism in a subset of proliferating cells
Source: Mol Syst Biol. 2025 Jun 5;21(8):4. doi: 10.1038/s44320-025-00099-0 (PMC12322234; doi:10.1038/s44320-025-00099-0)
Supplement: Supplementary file 11 — Expanded View Figures [file 44320_2025_99_MOESM11_ESM.pdf]

Expanded View Figures

Figure EV1. Pathway-centric metabolite ratios of all targeted metabolic pathways.

(**A**) Heatmap of scaled metabolite ratios by pathways covered in the targeted metabolomics approach. Ratios calculated for all metabolites of a specific pathway against a precursor metabolite. (**B**) Gap statistic, elbow method and silhouette method that determined the ideal number of clusters alongside K-means clustering based on Pearson's correlation coefficient method. (**C**) Clusters of cell lines from (**A**) appended with color coded legends for mutant status of common oncogenic drivers, culturing media conditions and tissue origins. Source data are available online for this figure.

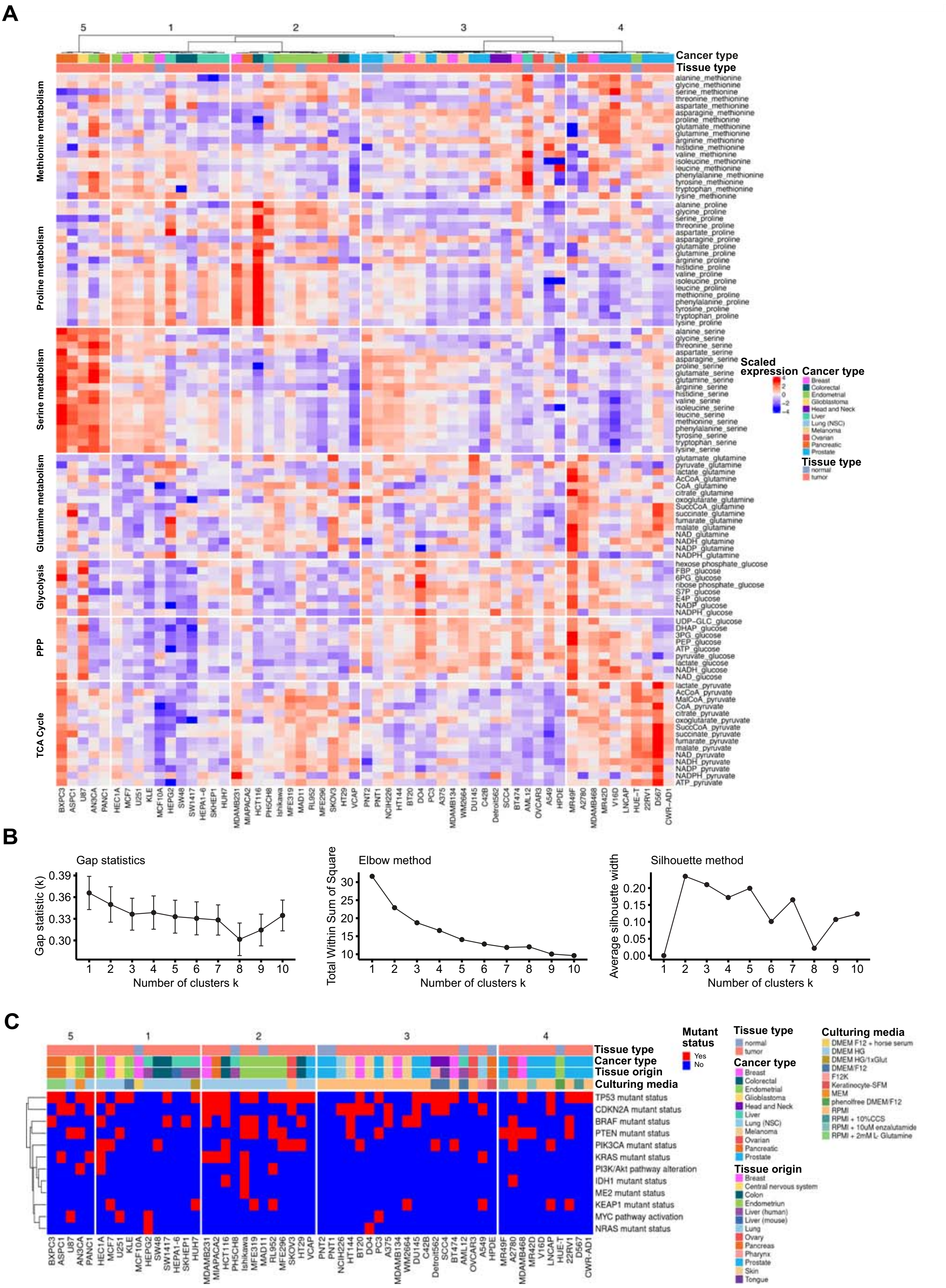
A
Cancer type
Tissue type
Methionine metabolism
Proline metabolism
Serine metabolism
Glutamine metabolism
Glycolysis
PPP
TCA Cycle
Scaled expression
Cancer type
Tissue type
B
Gap statistics
Gap statistic (k)
Number of clusters k
Elbow method
Total Within Sum of Square
Number of clusters k
Silhouette method
Average silhouette width
Number of clusters k
C
Tissue type
Cancer type
Tissue origin
Culturing media
Mutant status
Yes
No
TP53 mutant status
CDKN2A mutant status
BRAF mutant status
PTEN mutant status
PIK3CA mutant status
KRAS mutant status
PI3K/Akt pathway alteration
IDH1 mutant status
ME2 mutant status
KEAP1 mutant status
MYC pathway activation
NRAS mutant status

© The Author(s)

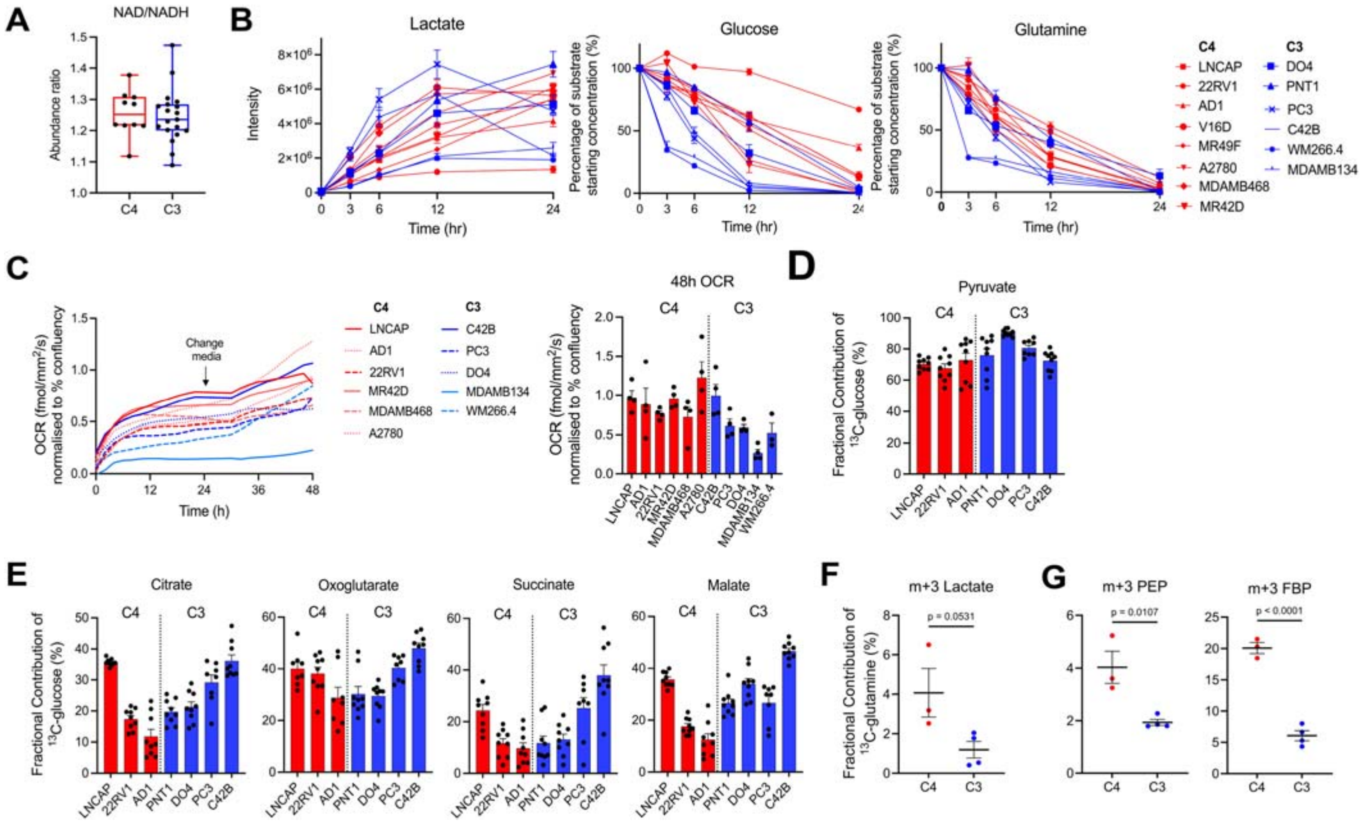

Figure EV2. Substrate preferences for cell line clusters identified by TCA cycle ratios.

(**A**) NAD+ to NADH ratio in cells of Clusters 4 and 3 (C4 $n = 10$ cell lines, C3 $n = 19$ cell lines, Unpaired student t-test, $p = 0.76$. The center of each box plot represents the median, the box boundaries correspond to the upper and lower quartiles, and the whiskers extend to the minimum and maximal values). (**B**) Lactate production and glucose and glutamine consumption over 24 h (C4 $n = 8$, C3 n-6, 3 biological replicates and 3 technical replicates per cell line, mean ± standard error of the mean). (**C**) Oxygen consumption rates (OCR) measured over 48 h, normalized to % confluency. Media changed at 24 h (left). OCR measurement for cell lines at 48 h (right) (C4 $n = 6$, C3 n-5, 3 biological replicates and 4 technical replicates per cell line). (**D**) Fractional contribution of [U-^{13}C]-glucose to intracellular pyruvate (C4 $n = 3$, C3 $n = 4$, 3 biological replicates and 3 technical replicates per cell line, mean ± standard error of the mean). (**E**) Fractional contribution of [U-^{13}C]-glucose to TCA metabolites citrate, oxoglutarate, succinate and malate (C4 $n = 3$, C3 $n = 4$, 3 biological replicates and 3 technical replicates per cell line, mean ± standard error of the mean). (**F**) Fractional contribution of [U-^{13}C]-glutamine to intracellular lactate (C4 $n = 3$, C3 $n = 4$, mean of 3 biological replicates and 3 technical replicates per cell line, p value vs Cluster 4 by Unpaired student's t-tests, mean ± standard error of the mean). (**G**) Fractional contribution of [U-^{13}C]-glutamine to m + 3 fructose 1,6-bisphosphate (FBP), and m + 3 phosphoenolpyruvate (PEP) (C4 $n = 3$, C3 $n = 4$, mean of 3 biological replicates and 3 technical replicates per cell line, p value vs Cluster 4 by Unpaired student's t-tests, mean ± standard error of the mean). Source data are available online for this figure.

© The Author(s)

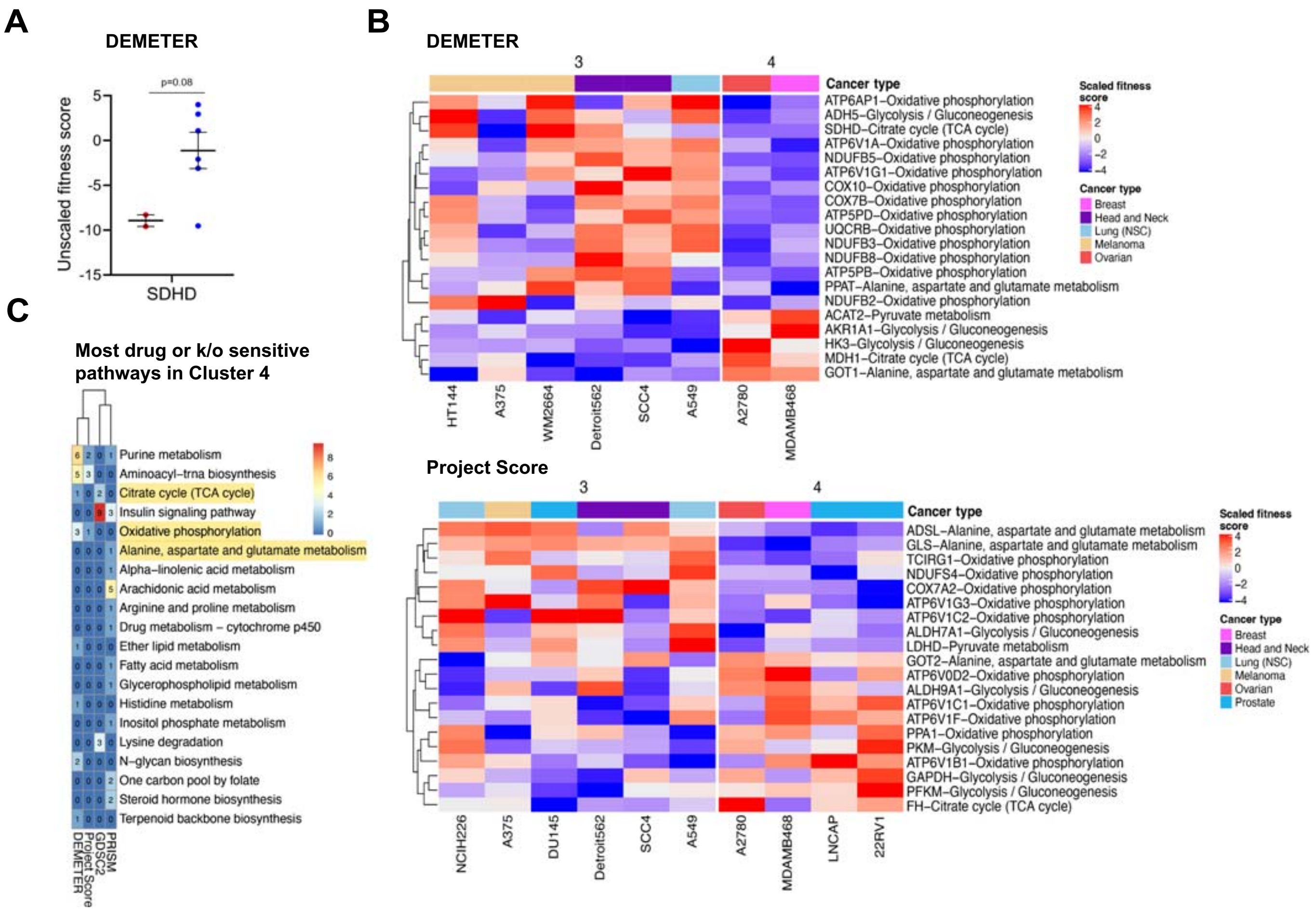

Figure EV3. Loss of function and drug database screen validation of pathway-centric metabolite ratio clusters.

(**A**) Genes related to the glucose utilization phenotype of C4 with trends of greater sensitivity to gene knockouts. (Unpaired student's t-test, ns $p > 0.05$, mean ± standard error of the mean, $n = 2$–6). (**B**) Top 20 gene knockouts and associated KEGG pathways with the greatest fitness scores between C4 and C3 in DEMETER and Project Score databases from glycolysis, TCA cycle, pyruvate metabolism, glycolysis/gluconeogenesis, and alanine, aspartate, and glutamate metabolism pathways. (**C**) Metabolic pathways associated with the top 20 drug and gene knockout sensitives for Cluster 4 for each database. Drugs and pathways matching C4 vulnerability model (yellow) are highlighted. Source data are available online for this figure.

 © The Author(s)

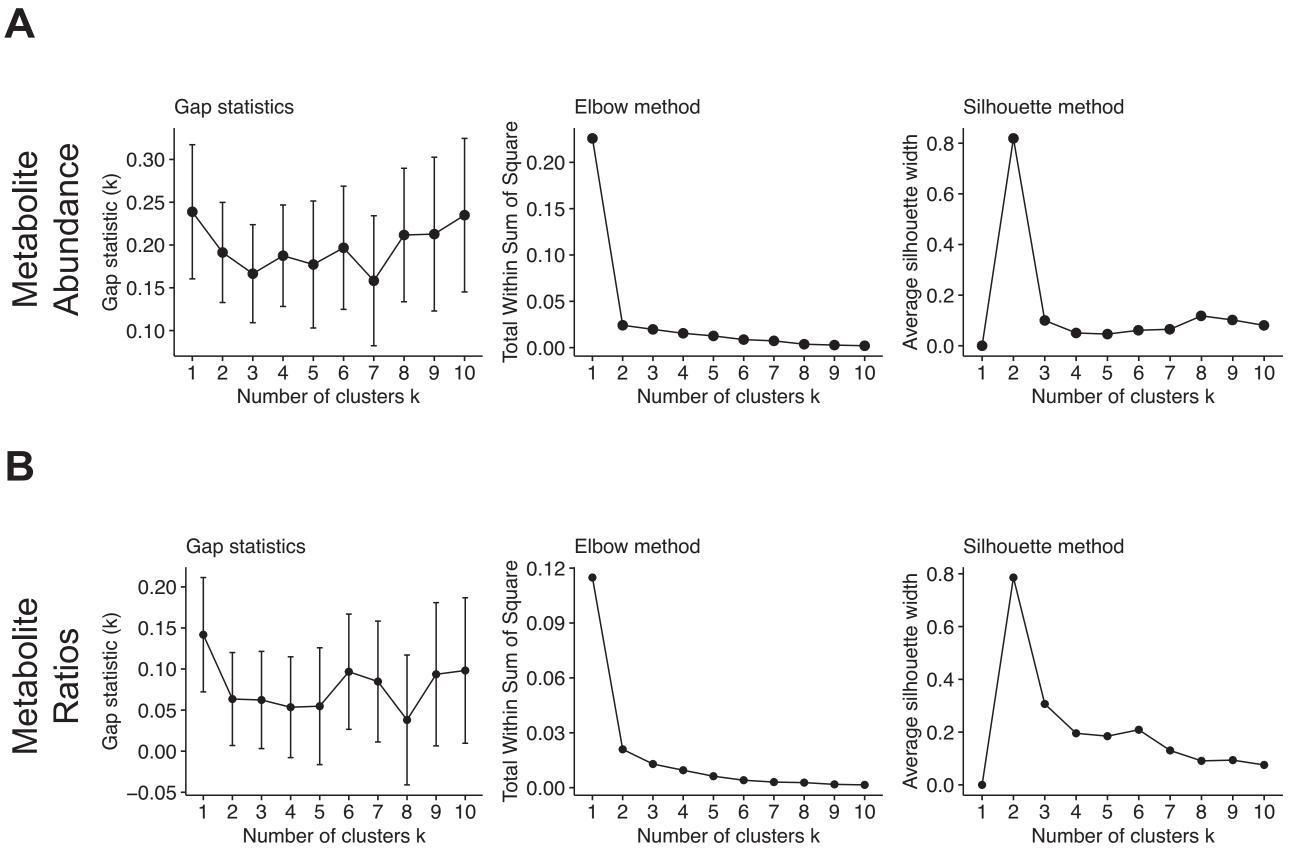

Figure EV4. Optimal cluster determination of metabolite and pathway-centric metabolite ratios using published data.

(**A**) The gap statistic (left), the total within sum of squares using the Elbow method (middle), and the average silhouette width for different number of clusters (right) for metabolite abundance-based clusters. (**B**) The gap statistic (left), the total within sum of squares using the Elbow method (middle), and the average silhouette width for different number of clusters (right) for pathway-centric metabolite ratio-based clusters. Source data are available online for this figure.

© The Author(s)
